# The Importance of Multimodality Imaging in the Diagnosis and Management of Patients with Infiltrative Cardiomyopathies: An Update

**DOI:** 10.3390/diagnostics11020256

**Published:** 2021-02-07

**Authors:** Radu Sascău, Larisa Anghel, Alexandra Clement, Mădălina Bostan, Rodica Radu, Cristian Stătescu

**Affiliations:** 1 Internal Medicine Department, “Grigore T. Popa” University of Medicine and Pharmacy, 700503 Iași, Romania; radu.sascau@gmail.com (R.S.); rodiradu@hotmail.com (R.R.); cstatescu@gmail.com (C.S.); 2 Cardiology Department, Cardiovascular Diseases Institute “Prof. Dr. George I.M.Georgescu”, 700503 Iași, Romania

**Keywords:** infiltrative cardiomyopathies, multimodality imaging, amyloidosis, sarcoidosis, hemochromatosis

## Abstract

Infiltrative cardiomyopathies (ICMs) comprise a broad spectrum of inherited and acquired conditions (mainly amyloidosis, sarcoidosis, and hemochromatosis), where the progressive buildup of abnormal substances within the myocardium results in left ventricular hypertrophy and manifests as restrictive physiology. Noninvasive multimodality imaging has gradually eliminated endomyocardial biopsy from the diagnostic workup of infiltrative cardiac deposition diseases. However, even with modern imaging techniques’ widespread availability, these pathologies persist in being largely under- or misdiagnosed. Considering the advent of novel, revolutionary pharmacotherapies for cardiac amyloidosis, the archetypal example of ICM, a standardized diagnostic approach is warranted. Therefore, this review aims to emphasize the importance of contemporary cardiac imaging in identifying specific ICM and improving outcomes via the prompt initiation of a targeted treatment.

## 1. Introduction

Cardiomyopathies represent a heterogeneous group of primary myocardial disorders and, most importantly, a leading cause of heart failure [1]. On a phenotypic basis, three major types of cardiomyopathy have been described: hypertrophic, dilated, and restrictive cardiomyopathies (RCMs). RCM is the least common of the three main forms and is further classified as infiltrative and noninfiltrative, storage diseases, and endomyocardial disorders [2].

Infiltrative cardiomyopathies (ICMs) comprise a broad spectrum of inherited and acquired conditions, where the progressive buildup of abnormal substances within the myocardium results in left ventricular hypertrophy and manifests as restrictive physiology [3]. We focused primarily on cardiac amyloidosis, sarcoidosis, and hemochromatosis, although the other etiologies, such as Fabry disease, Dannon disease, and Friedreich’s ataxia, are also still important, but less common.

Cardiac amyloidosis, the paradigm of RCM, is the result of amyloid deposition in the heart, which commonly occurs in immunoglobulin-light chain amyloid (AL) amyloidosis and transthyretin amyloid (ATTR) amyloidosis (either hereditary or wild-type) [4]. In AL amyloidosis, the most frequent type of systemic amyloidosis, excessive monoclonal light chain production, secondary to plasma cell dyscrasia, promotes amyloid fibrillogenesis. By comparison, in ATTR amyloidosis, transthyretin, a homotetrameric transport protein produced by the liver, dissociates into monomers that subsequently misfold and self-assembly in amyloid fibrils [5,6].

Sarcoidosis is a multisystem, inflammatory, noncaseating granulomatous disease of unknown etiology that mostly affects the lungs and lymphatic nodes. The disorder presents three sequential histological stages: edema, granulomatous infiltration, and fibrosis, the latter stage being not only the most severe but also an independent prognostic factor [7,8]. Cardiac sarcoidosis accounts for substantial morbi-mortality, with reported mortality rates higher than 60% [9,10]. Furthermore, according to a recent CMR imaging study performed on 205 patients with sarcoidosis, the prevalence of cardiac involvement seems to exceed 25%, and thus, there is a need for awareness and expertise in diagnosing ICMs [11,12].

Hemochromatosis may result either from a gene mutation (hereditary hemochromatosis) or from a secondary cause (hemosiderosis) [7,13]. Hereditary hemochromatosis (HH) is marked by iron overload due to enhanced gastrointestinal absorption. Cardiac involvement appears later when compared to other organs engaged in the pathogenic process (e.g., liver, pancreas, skin) and is less common, occurring only in 15–20% of cases [14]. Disproportionate iron accumulation within the heart determines, in the early stage, impaired left ventricular (LV) diastolic function, with a restrictive filling pattern that, in the absence of iron chelation therapy, invariably progresses to LV dilatation and altered systolic function [15].

Noninvasive multimodality imaging has gradually eliminated endomyocardial biopsy from the diagnostic workup of infiltrative cardiac deposition diseases. However, even with modern imaging techniques’ widespread availability, these pathologies persist in being largely under- or misdiagnosed. Considering the advent of novel, revolutionary pharmacotherapies for cardiac amyloidosis, the archetypal example of ICM, and a life-threatening condition, a standardized diagnostic approach is warranted. Therefore, this review aims to emphasize the importance of contemporary cardiac imaging in identifying specific ICM and improving outcomes via the prompt initiation of a targeted treatment.

## 2. Conventional Transthoracic Echocardiography (TTE)

Transthoracic echocardiography (TTE) is a powerful, widely available diagnostic tool and a critical initial step in assessing patients with ICMs. It provides valuable information regarding LV wall thickness, LV cavity size, LV systolic and diastolic function, the presence of pericardial effusion or right heart involvement. Echocardiographic findings play a pivotal role in recognizing ICMs, but they only arouse suspicion about the etiology. TTE is considered neither specific nor sensitive in diagnosing cardiac amyloidosis [16]. However, according to a sizeable multicentric study performed by Boldrin M et al., echocardiography’s diagnostic performance in patients with proven systemic AL amyloidosis can be improved through the use of highly sensitive and specific cutoffs [17]. Likewise, a normal echocardiogram cannot rule out cardiac involvement in a patient with systemic sarcoidosis [18]. Additionally, TTE is considered a second-line imaging method, after cardiac magnetic resonance, for the evaluation of cardiac hemochromatosis, being more useful in regular clinical follow-up and screening than in quantifying biventricular systolic function [15,19,20].

### 2.1. Left Ventricular Wall Thickness and Cavity Size

#### 2.1.1. Left Ventricular Wall Thickness and Cavity Size in Amyloidosis

As the infiltrative process dominantly involves the left ventricular walls, the typical echocardiographic findings in cardiac amyloidosis consist of a concentric LV pseudohypertrophy with a small LV cavity size, elements that can be seen in more than 90% of cases [5]. The hallmark of cardiac amyloidosis is the mismatch between low QRS voltage on the electrocardiogram and increased LV wall thickness on the echocardiogram [4]. However, up to 20% of patients with cardiac amyloidosis can have electrocardiographic evidence of LV hypertrophy, especially those with ATTR wild-type amyloidosis [21]. ATTR wild-type amyloidosis is marked out by greater LV wall thickness and mass when compared to hereditary ATTR amyloidosis and AL amyloidosis, indicating a longer time of amyloid accumulation [22]. The sparkling or granular appearance of the hypertrophied myocardium, initially viewed as a distinctive feature of cardiac amyloidosis, is not a diagnostic sign, as it lacks sensitivity [10]. Nevertheless, in the early stages, asymmetrical septal hypertrophy with LV outflow tract obstruction can be noticed [15]. The propensity for symmetrical hypertrophy is more pronounced in AL amyloidosis, while in ATTR amyloidosis, there is a tendency towards an asymmetrical increase in LV wall thickness [23].

#### 2.1.2. Left Ventricular Wall Thickness and Cavity Size in Sarcoidosis

The echocardiographic reports in cardiac sarcoidosis are of significant variability. A mild increase in left ventricular wall thickness, imitating hypertrophic cardiomyopathy, can be encountered during edema and granulomatous infiltration [24]. Conversely, basal septal thinning, which is highly prevalent and a characteristic feature of cardiac sarcoidosis, is the consequence of myocardial fibrosis [7,25]. LV cavity size is relatively normal or increased [7].

#### 2.1.3. Left Ventricular Wall Thickness and Cavity Size in Hemochromatosis

Myocardial concentric or asymmetric nonextreme hypertrophy with gradual left ventricular remodeling and dysfunction is the commonest phenotype in cardiac hemochromatosis [26]. Rozwadowska et al. performed an analysis on 39 volunteers with early diagnosed HH and 19 with long-lasting and long-treated HH and concluded that both early and old HH are linked to LV hypertrophy, independent of other comorbidities such as arterial hypertension and diabetes [27].

### 2.2. Diastolic Dysfunction

#### 2.2.1. Diastolic Dysfunction in Amyloidosis

Diastolic function is dramatically affected in cardiac amyloidosis, the degree of impairment being related to the extent of amyloid infiltration and an independent predictor of poor outcomes and mortality [28]. As the disease progresses, LV wall stiffness increases, leading to impaired relaxation and, later, to high filling pressures. A restrictive filling pattern, the paradigm of ICMs, is outlined by high peak E wave velocity with short E wave deceleration time, decreased atrial filling velocity (A wave) with increased E/A ratio. The alteration of diastolic function, together with amyloid atrial infiltration, leads to biatrial enlargement and increases thromboembolic risk [23]. Intra-atrial thrombosis is common even among patients with cardiac amyloidosis and sinus rhythm, as reported by a large autopsy study [29]. In advanced cardiac amyloidosis, the pulmonary vein flow pattern is abnormal, with reduced systolic flow velocity and increased diastolic flow velocity [30].

**Figure 1:**
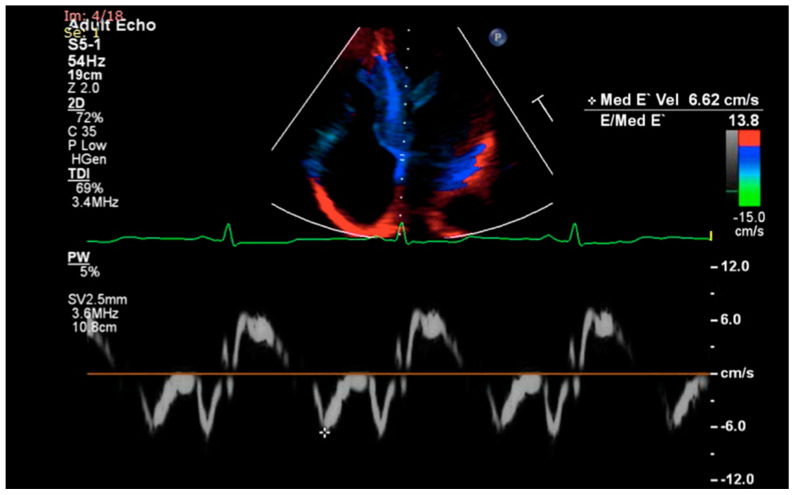
Tissue Doppler imaging demonstrating decreased septal e’ velocity and increased E/e’ ratio in a female with cardiac amyloidosis.

Tissue Doppler imaging (TDI) is a relatively new echocardiographic technique that helps to distinguish between cardiac amyloidosis and constrictive pericarditis or hypertrophic cardiomyopathy. Early diastolic mitral annulus velocity (e’) as assessed by TDI incrementally decreases during the course of cardiac amyloidosis, while in constrictive pericarditis and hypertrophic cardiomyopathy it is normal or only slightly reduced (Figure 1) [31]. In addition, E/e’ ratio values appear to be higher in cardiac amyloidosis than in hypertensive LV hypertrophy and enhance echocardiography’s discrimination power when combined with LV basal longitudinal strain assessment [32].

#### 2.2.2. Diastolic Dysfunction in Sarcoidosis and Hemochromatosis

Analogously, diastolic dysfunction (DD) is consistently present in hemochromatosis and sarcoidosis. It starts as grade 1 DD or impaired relaxation and it ends with grade 3 DD or restrictive pattern with elevated LV filling pressures [15,24].

In hemochromatosis, a pseudonormal filling pattern, also known as grade 2 DD, can be encountered as well, and can be better characterized with tissue Doppler imaging, which generally reveals decreased diastolic early filling mitral annular tissue velocity, beginning with the initial stages of the disease. An increase in the duration of the pulmonary venous atrial reversal flow is of additional help. With regard to the connection between DD and outcomes in myocardial iron overload cardiomyopathies, very few data are available. Restrictive DD can lead to the emergence of right-sided heart failure with preserved LV ejection fraction [12,15].

In cardiac sarcoidosis, the degree of LV and/or RV DD appears to closely connect with the extent of myocardial involvement evaluated via cardiac magnetic resonance imaging [12,15]. Notably, DD does not represent a specific sign of cardiac involvement in sarcoidosis, despite being invariably present [10].

### 2.3. Systolic Function

#### 2.3.1. Systolic Function in Amyloidosis

In cardiac amyloidosis, longitudinal ventricular contraction impairment emerges before the deterioration of radial contraction. Therefore, the LV ejection fraction (LVEF) as determined by 2D conventional TTE, tends to be preserved until advanced stages, and it is not a reliable measure of LV systolic performance. The mitral annulus’s systolic excursion in M-mode echocardiography (MAPSE) is often reduced, despite normal LVEF [23].

Myocardial performance index (MPI) or Tei index, defined as the sum of isovolumetric contraction time and isovolumetric relaxation time divided by ejection time, increases in cardiac amyloidosis. This finding was firstly described by Tei et al., who proved that in cardiac amyloidosis, the isovolumetric relaxation time is prolonged in all stages of the disease. Accordingly, the pre-ejection time is prolonged, and the ejection time is reduced, leading to an increase in MPI [30,33].

#### 2.3.2. Systolic Function in Sarcoidosis

Due to regional wall motion abnormalities that do not follow a coronary distribution, systolic dysfunction is a common finding in cardiac sarcoidosis [15,34]. Patients with cardiac sarcoidosis and LVEF < 50% are considered to have inferior survival rates than those with preserved ejection fraction and to be less responsive to corticosteroid treatment [35].

#### 2.3.3. Systolic Function in Hemochromatosis

In cardiac hemochromatosis, if the cause of iron overload is not corrected, most of the subjects develop LV remodeling with dilation and reduced LVEF [36].

Echocardiography aids diagnosing LV systolic dysfunction, but this finding does not specifically suggest cardiac hemochromatosis. There is little information regarding the time path of progressing to a dilated phenotype from a restrictive one, as well as the factors that promote this occurrence. Nevertheless, advanced echocardiographic techniques, including strain rate imaging, deliver a more accurate characterization of LV systolic performance in cardiac hemochromatosis [12].

### 2.4. Right Heart Involvement

#### 2.4.1. Right Heart Involvement in Amyloidosis

**Figure 2:**
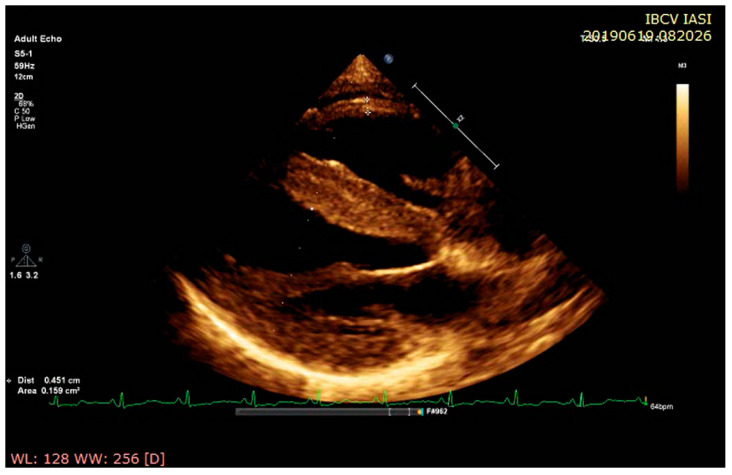
Echocardiographic assessment at rest in a young female with ATTR amyloidosis: parasternal long-axis view demonstrating concentric left ventricular pseudohypertrophy with concomitant right ventricular free wall hypertrophy.

Concomitant right ventricular (RV) free wall hypertrophy strongly suggests cardiac amyloidosis or another ICM [37]. When the right ventricle is caught up in the insoluble amyloidotic fibrils’ pathological deposition, the anterior wall is primarily affected [30] (Figure 2). RV involvement occurs after LV amyloid infiltration, and it essentially worsens the prognosis [37]. Longitudinal RV systolic function impairment leads to a reduction in the tricuspid annular plane systolic excursion (TAPSE) in M mode echocardiography [22]. A cutoff <14 mm of TAPSE independently predicted the risk of major adverse cardiac events defined as death, heart transplantation, and acute heart failure in 82 patients with confirmed cardiac amyloidosis [38]. The prognostic and predictive value of RV involvement in cardiac amyloidosis was also evaluated in a recent retrospective study, published by Cicco et coworkers. They retrospectively evaluated a total of135 patients with systemic amyloidosis, 54 of whom had signs of cardiac amyloidosis (CA) at baseline. As a control group, they included 81 patients with non-cardiac amyloidosis (nCA). The authors found a significant increase in the right atrium, right ventricular basal diameter, and the diameter of the inferior cava vein at rest in CA patients. They also observed statistically significant differences in right ventricular wall thickness, which was increased in CA patients (CA 9.87 ± 1.73 vs. nCA 7.00 ± 1.05 mm; *p* = 0.0001). Regarding the RV systolic function, evaluated by measurement of the tricuspid annular plane excursion, they found a reduced value of TAPSE in CA compared to nCA patients (CA 18.73 ± 8.32 vs. nCA 26.58 ± 1.73 mm; *p* = 0.0317). Although elevated values of estimated pulmonary arterial pressure have been observed in CA patients (CA 38.27 ± 10.67 vs. nCA 28.38 ± 3.75 mmHg; *p* = 0.0053), there were no differences regarding the tricuspid regurgitation velocity between the two groups. At the same time, a decreased TAPSE indicated a worse prognosis and displayed a significant positive correlation with lymphocyte count, gamma globulins, monoclonal components, and immunoglobulin G values, while the inferior vena cava diameter, the right atrium area, and the estimated pulmonary arterial pressure correlated only with diastolic function, evaluated as E/e’ ratio [39].

In order to compare the right ventricular involvement in transthyretin amyloidosis and hypertrophic cardiomyopathy, Arvidsson et al. evaluated the echocardiographic conventional parameters and the RV global and segmental strain of 42 subjects with ATTR amyloidosis and echocardiographic evidence of left ventricular hypertrophy (cardiac ATTR), 19 ATTR subjects with normal LV wall thickness (non-cardiac ATTR), 25 patients with biopsy proven or genetically diagnosed HCM, and 30 healthy volunteers. They emphasized that, regarding RV structure and function, only RV segmental strain may help in distinguishing between these two pathologies. In the cardiac ATTR amyloidosis subgroup, an apex-to-base RV strain gradient was noticed, with relative apical sparring, analogously to what has been previously reported for the LV. Conversely, segmental RV strain was lower in the apical region of HCM subjects; this reverse pattern warrants more research [40]. Concerning RV diastolic function, equivalent modifications in the tricuspid inflow pattern are generally detected. RV dilatation reflects advanced disease and portends a poor prognosis [38].

#### 2.4.2. Right Heart Involvement in Sarcoidosis

Even though the biggest predilection for granulomatous infiltration is located within the basal interventricular septum and LV lateral free wall, it is essential to acknowledge that cardiac sarcoidosis can directly impact the RV [24]. Half of the individuals with cardiac sarcoidosis manifest RV wall motion abnormalities and systolic dysfunction [41]. LV and lung sarcoid infiltration can result in pulmonary hypertension and RV dilatation [42]. The true prevalence of pulmonary hypertension in sarcoidosis is unknown, ranging from 5 to 20% to more than 50% in symptomatic subjects with persistent or unexplained dyspnea [43].

Cardiac sarcoidosis and arrhythmogenic right ventricular cardiomyopathy (ARVC) can have overlapping phenotypes. In an analysis performed by Philips et al., 15 subjects recorded in the Johns Hopkins ARVC registry were afterward reclassified as having cardiac sarcoidosis [44].

RV abnormalities can be better characterized by cardiac magnetic resonance (CMR) imaging, with RV involvement in cardiac sarcoidosis being an important prognostic factor and a valuable risk stratification tool [45,46].

#### 2.4.3. Right Heart Involvement in Hemochromatosis

As hemochromatosis progresses, RV dilation and systolic dysfunction can become detectable [14].

### 2.5. Other Echocardiographic Findings

#### 2.5.1. Other Echocardiographic Findings in Amyloidosis

**Figure 3:**
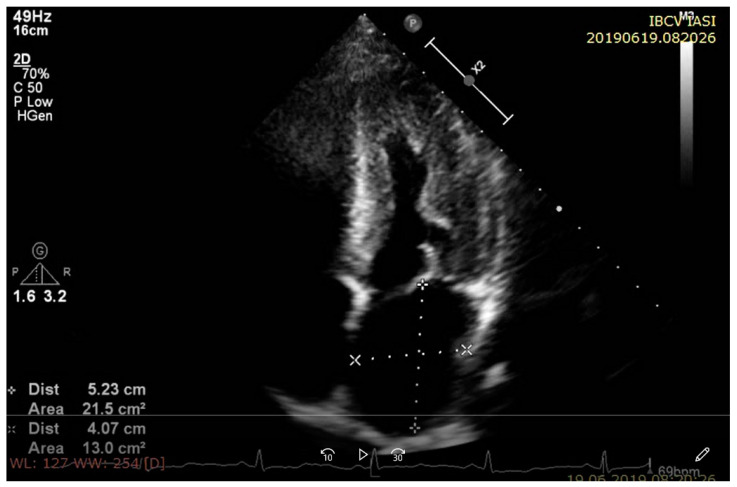
Transthoracic echocardiogram, apical four-chamber view in a patient with cardiac amyloidosis showing: increased thickness of left ventricle wall with “granular sparkling” appearance, increased biatrial dimensions in contrast with a small left ventricle cavity, and pericardial effusion.

Although the majority of the echocardiographic signs of cardiac amyloidosis are nonspecific, they can become highly suggestive when integrated into the context. Biatrial enlargement, small pericardial and pleural effusions, and thickening of the atrioventricular valve and of the interatrial septum are other echocardiographic features of cardiac amyloidosis [21] (Figure 3). Zhao et al. evaluated the prognostic value of left atrial (LA) size on 104 subjects with cardiac amyloidosis. According to their study, LA enlargement, defined as a LA diameter indexed to the body surface area greater than 23 mm/m^2^, is strongly associated with all cause-mortality and severe heart failure [47]. The study conducted by Falk et al. was among the earliest echocardiographic studies and showed that increased atrial septal thickening was present in 60% of the subjects with cardiac amyloidosis and had 60% sensitivity and 100% specificity for cardiac amyloidosis diagnosis when combined with enhanced myocardial echogenicity [48,49]. Left heart valve thickening is frequent in AL amyloidosis and linked to worse outcomes and higher mortality [50]. Damy T et al. conducted an observational study on 266 patients with cardiac amyloidosis and proved that pericardial effusion, when present, even in small amounts, is a strong predictor of death [51]. Of note, no differences in subtype-specific causes of death were noted between AL and ATTRwt cardiac amyloidosis patients [52].

#### 2.5.2. Other Echocardiographic Findings in Sarcoidosis

In cardiac sarcoidosis, granulomatous deposition can be located in any cardiac structure, including the endocardium, myocardium, pericardium, conduction system, coronary arteries, and vena cava [10]. Myocardial granulomatous infiltration can cause LV aneurysm formation. Mitral regurgitation is the result of either papillary muscle malfunction or direct infiltration of the valve [12]. Pericardial effusion is a rare manifestation of cardiac sarcoidosis, but it has been reported in various case reports [53].

Additionally, cardiac sarcoidosis can mimic coronary artery disease, Takotsubo cardiomyopathy, right ventricular cardiomyopathy, hypertrophic cardiomyopathy, and valvular dysfunction [40]. The bright echogenicity of the interventricular septum and the free wall indicates scars and inflammation [24].

#### 2.5.3. Other Echocardiographic Findings in Hemochromatosis

In cardiac hemochromatosis, echocardiographic findings detect the repercussion of iron overload, which vary from a restrictive pattern with biatrial enlargement to biventricular dilatation with systolic dysfunction. A myocardial phenotype of left ventricular noncompaction may be present in cardiac hemochromatosis [26].

## 3. Advanced Echocardiographic Techniques

### 3.1. Advanced Echocardiographic Techniques in Amyloidosis

In cardiac amyloidosis, TDI enables the distinction between cardiac amyloidotic pseudohypertrophy and true hypertrophy. Myocardial velocities recorded by placing the sample volume in the septal or lateral ventricular myocardium, adjacent to the mitral annulus, are considerably reduced as the amyloidotic infiltration of the heart compromises myocytes’ longitudinal contraction. If TDI recognizes several limitations, such as the interference with translational and tethering movements of the heart, speckle tracking echocardiography (STE) possesses excellent spatial and temporal resolution [30].

STE is a valuable diagnostic tool, capable of differentiating cardiac amyloidosis from hypertensive heart disease and hypertrophic cardiomyopathy. A simple diagnostic algorithm, based on T troponin blood levels and two echo-derived strain parameters, namely global longitudinal strain and apical to basal longitudinal strain gradient, was recently proposed as an alternative for diagnosing cardiac involvement in AL amyloidosis, displaying greater accuracy when compared to consensus criteria [30].

**Figure 4:**
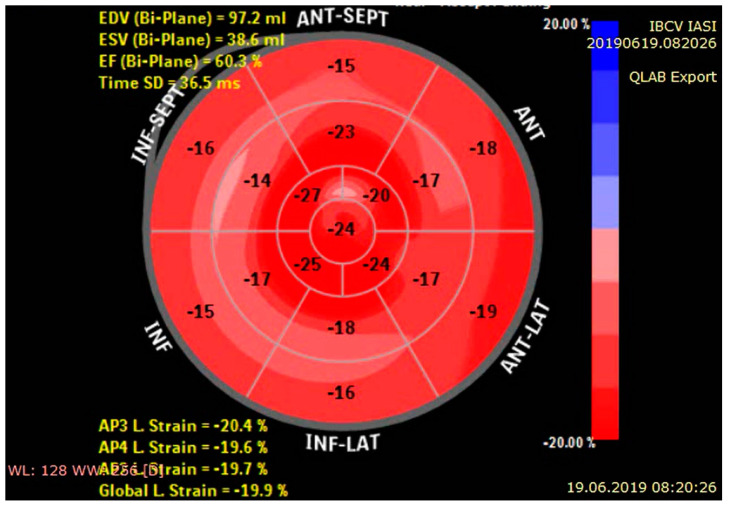
Distinctive bull′s-eye plot in cardiac amyloidosis, with slight impairment of the global longitudinal strain (GLS) in the basal and mid-ventricular segments and relative apical-sparing.

Quarta et al. performed a detailed echocardiographic study on 172 subjects with amyloidosis and highlighted that global longitudinal strain values are significantly reduced even in patients with preserved ejection fraction [54]. The abnormal global longitudinal strain has an incremental prognostic value, connected with poor survival in both types of cardiac amyloidosis [22]. However, longitudinal myocardial deformation is mainly reduced in the mid and basal segments, the apical sparing of the longitudinal strain generating a distinctive “bulls-eye pattern” described as the “cherry-on-the-top” sign. This singular conservation of the apex’s contractile function mainly reflects a less extent of amyloid accumulation at the ventricular apex than at the base [4] (Figure 4). Pagourelias et al. suggested that a cut off value of 4.1 of LVEF strain ratio (LVEF/absolute value of global longitudinal strain) facilitates the identification of cardiac amyloidosis within the group of thickened myocardium pathologies, being particularly useful in the subset of patients with mild hypertrophy and preserved ejection fraction [55]. Notably, not only is longitudinal strain impaired in cardiac amyloidosis, but also circumferential and radial deformations [5].

Despite the extensive literature on LV longitudinal strain as assessed via speckle tracking echocardiography and tissue Doppler imaging, it is worth mentioning that RV free wall longitudinal strain (FWLS) has also emerged as a powerful prognostic tool in cardiac amyloidosis. According to the results of a retrospective analysis on 36 subjects with AL amyloidosis and 57 with ATTR amyloidosis, baseline RV FWLS was independently associated with all-cause mortality and cardiovascular hospitalizations at 1 year follow-up, whereas other echocardiographic parameters of right heart performance were not. RV FWLS decreased significantly during follow-up echocardiographic evaluations, in both types of amyloidosis, whereas other RV measurements did not, designating RV FWLS as a sensitive measure of the extent of RV involvement in cardiac amyloidosis [56]. A decrease in RV free wall systolic velocity via tissue Doppler echocardiography can be recorded as well [10].

Left atrial strain, as assessed by 2D- speckle tracking echocardiography (STE), came to light as a new useful marker for confirming cardiac amyloidosis. All left atrial strain and strain rate parameters seem to be significantly reduced in cardiac amyloidosis subjects, to a greater extent than in the hypertensive population. A LA reservoir strain cut-off value of 20% had a sensitivity of 86.4% and a specificity of 88.6% in detecting cardiac amyloidosis in a study that has included 44 patients with cardiac amyloidosis and 25 with hypertensive heart disease and increased LV wall thickness [57]. Moreover, in cardiac amyloidosis, 2D-STE-derived LA phasic functions strongly correlate with LV global longitudinal strain. Nochioka et al. showed that worse LA strain in cardiac amyloidosis is linked to a more significant impairment of LV systolic and diastolic functions [58].

The research conducted by Mohty et al. was the first to investigate the relationship between LA strain parameters derived by three-dimensional STE, NT-proBNP, and cardiac troponin T levels and Mayo Clinic staging on a cohort comprising 77 subjects with confirmed AL amyloidosis and 39 healthy controls. Decreased 3D-LA total emptying fraction and 3D peak atrial longitudinal strain were associated with a reduced two-year survival rate, independently of LA volume [59].

### 3.2. Advanced Echocardiographic Techniques in Sarcoidosis

As mentioned above, TTE has low sensitivity in diagnosing cardiac sarcoidosis, the emergence of newer imaging techniques leading to an improvement in the diagnostic performance [14]. Myocardial deformation strain imaging helps to detect subclinical cardiac sarcoidosis in the context of reduced global longitudinal strain (GLS) with preserved LVEF [24].

A single-center retrospective study, including 83 patients with extracardiac, biopsy-proven sarcoidosis and definite/probable cardiac involvement, evaluated the diagnostic and prognostic values of 2D-STE in cardiac sarcoidosis. Abnormal LV GLS, inferoseptal and inferior wall strain, and RV GLS are markers of cardiac sarcoidosis, even when LVEF and RV systolic parameters are within normal ranges. Regarding STE’s predictive value in cardiac sarcoidosis, in this analysis, a LV GLS more positive than −14% was linked to a higher rate of hospitalizations and heart failure [60].

Kusunose K et al. prospectively evaluated with 2D-STE 139 patients with systemic sarcoidosis without preexisting structural heart disease and 52 age- and gender-matched control subjects. They revealed that basal longitudinal strain and RV free wall longitudinal strain provide incremental value over standard evaluation, being significantly impaired in patients with cardiac sarcoidosis development than in those without. Subjects with biventricular strain alteration also had a shorter event-free survival [61].

In addition, a meta-analysis performed by Barssoum K. et al., comprising 9 studies and 967 subjects, proved that both LV GLS and global circumferential strain are substantially lower in extracardiac sarcoidosis subjects and no clinical evidence of cardiac involvement and that the degree of LV GLS impairment correlates with the occurrence of major cardiac events [62]. Additionally, the circumferential strain has proved to be an indicator of fibrosis burden in subjects with extracardiac sarcoidosis. Reduced circumferential strain via 2D-STE correlates with extensive delayed enhancement in cardiac magnetic resonance imaging, as demonstrated by Orii et al.’s study [63].

### 3.3. Advanced Echocardiographic Techniques in Hemochromatosis

In hereditary hemochromatosis cardiomyopathy, TDI is useful in emphasizing a reduction in early diastolic velocity of the lateral and medial mitral annulus [64]. According to a study conducted by Shizukuda et al., myocardial strain imaging appears to correlate better with the impact of oxidative stress on LV diastolic function than with iron overload [65].

It appears that 2D-STE is more sensitive in detecting cardiac involvement in HH compared to conventional transthoracic echocardiography. Rozwadowska K et al. prospectively recruited 24 subjects with early diagnosed HH and without structural heart disease and 23 age- and sex-matched healthy volunteers. All standard echocardiographic measurements were within normal limits in all study subjects. However, 2D-STE demonstrated remarkably worse basal and apical rotation, twist, and torsion values in the HH subgroup, with decreased peak systolic longitudinal strain [66].

Byrne D et al. demonstrated that in 25 patients with newly diagnosed HH and without evidence of heart failure, radial strain assessed via STE, left atrial force calculated by the Manning method, and isovolumic relaxation time substantially improved after one year of treatment with venesection. These results indicate that radial strain, isovolumic relaxation time, and left atrial force are valuable in monitoring cardiac function, guiding the intensity of treatment, and detecting subclinical cardiac involvement [67].

## 4. Computed Tomography Imaging

Computed tomography (CT) imaging is entirely appropriate for describing the key structural characteristic of RCM and, therefore, of ICM, such as biatrial enlargement, dilatation of inferior vena cava and coronary sinus, pericardial effusion, pulmonary congestion, and pleural effusion. It is a powerful instrument for quantifying LV mass and wall thickness when other imaging techniques are not available or are contraindicated and also helps detect extracardiac involvement in ICM (e.g., pulmonary nodules and fibrosis with lymphadenopathy in sarcoidosis). Its primary disadvantage consists of high radiation exposure [15].

### 4.1. Computed Tomography Imaging in Amyloidosis

Improvements in CT imaging technique have extended the usability of cardiac CT, initially designed with the purpose of assessing the coronary arteries. Nowadays, it allows myocardial characterization via late iodine enhancement (LIE) imaging as well as extracellular volume (ECV) quantification [68]. Treibel T et al. suggested that CT can be used to appreciate cardiac amyloid burden and that ECV assessed by CT correlates well with the myocardial ECV obtained through CMR imaging. These authors performed a 5 min contrast-enhanced gated cardiac CT analysis on 26 patients with a biopsy-proven systemic amyloidosis and 26 patients with severe aortic stenosis and proved that ECV was higher among the CA subgroup [69].

Of note, the association between aortic stenosis and cardiac amyloidosis is quite common, especially among the elderly. The differentiation of these two pathologies is challenging, as aortic stenosis and cardiac amyloidosis exhibit some identical features. The aortic valve calcium score assessed by non-contrast CT helps to grade aortic stenosis severity [70]. Supplementarily, the assessment of myocardial ECV during transcatheter aortic valve replacement (TAVR) planning CT can discover occult, concomitant cardiac amyloidosis in patients with severe aortic stenosis [71]. Equally importantly, in systemic amyloidosis, CT imaging can identify lung and respiratory tract involvement (e.g., nodular pulmonary amyloidosis, diffuse alveolar-septal amyloidosis, tracheobronchial amyloidosis, or amyloidosis of the pleura) [72].

### 4.2. Computed Tomography Imaging in Sarcoidosis

In systemic sarcoidosis, CT imaging is useful in recognizing both cardiac and lung involvement. Typical pulmonary CT findings in sarcoidosis encompass mediastinal and symmetric hilar lymphadenopathy and upper lobe predominant perilymphatic pulmonary nodules [73]. The ability of contrast-enhanced CT imaging to characterize myocardial tissue beyond its structure was also underlined by Raimondi et al., who reported a case of cardiac sarcoidosis in which marked myocardial hypoattenuation on CT imaging was in concordance with CMR, PET, and SPECT imaging findings [74].

In cardiac sarcoidosis, extensive myocardial scarring enhances the risk of malignant ventricular arrhythmia and sudden cardiac death and, thus, the importance of its noninvasive assessment. It is commonly known that late gadolinium enhancement imaging with CMR enables the visualization of myocardial fibrosis. However, as mentioned above, a new functional CT technique was recently developed and allows the quantitative evaluation of myocardial perfusion and ECV. In this framework, So A et al. pointed out that this CT imaging may have an additive value to CMR or PET to diagnose cardiac sarcoidosis, being particularly useful in subjects with metallic implants and cases where CMR or PET are not available or are contraindicated [75].

### 4.3. Computed Tomography Imaging in Hemochromatosis

There is limited literature regarding cardiac computed tomography’s benefits in evaluating cardiac hemochromatosis, particularly in assessing cardiac iron levels. CMR imaging is the gold-standard technique for the evaluation of cardiac hemochromatosis, but cardiac CT can be a useful method of evaluating cardiac function [20].

## 5. Cardiac Magnetic Resonance Imaging

CMR has high accuracy in assessing the cardiac interstitium and, therefore, ICM. The most used IRM evaluation methods are static images, cine and contrast-enhanced imaging, and parametric mapping. The static images have the role of differentiating according to the tissue characteristics, the pericardial myocardium, and the vascular structures, and T1 and T2 add complementary information. T1 weighted images show a high signal from fat, and T2 weighted short tau inversion recovery (STIR) images can identify myocardial edema, as is the case of acute sarcoidosis. It is possible to use averaged heartbeats typical cine CMR images to provide a better temporal resolution. However, real-time images have additive value, as in the case of showing the typical septal shift in the context of respiratory maneuvers to objectify the restrictive pattern of the atrioventricular valves [76].

Late gadolinium enhancement (LGE) is an essential feature of CMR and can differentiate fibrosis, scarring, and myocardial infiltration, thus contributing to the differentiation between different types of ICM. The role of LGE is a diagnostic one and a prognostic one [77]. Parametric mapping methods help in quantitative measurements, but also in tissue characterization. Recent studies used T1 mapping to quantify inflammation and myocardial fibrosis, as well as to measure native T1 relaxation times, parameters that are correlated with the severity of amyloidosis, for example, a fact that proves its prognostic role [78]. The combined use of native and post-contrast T1 mapping allows the evaluation of myocardial extracellular volume fraction, an element of differentiation between different amyloidosis types.

In ICM, although the evaluation of ventricular walls often shows an increased thickness by measuring steady-state free precession sequences, the major advantage of using CMR lies in the ability of this imaging method to characterize the myocardial tissue in terms of its intrinsic magnetic characteristics and distribution pattern of the gadolinium-based contrast agents [2].

Myocardial edema and inflammation appear as hyperintense areas in fast spin-echo T2-weighted sequences or quantitative T2 mapping, while decreased T2-weighted signal intensities are most commonly associated with iron overload myocardial disorders [79]. Nearly one-third of restrictive cardiomyopathy cases have delayed myocardial enhancement due to inflammation and associated fibrosis [80]. At the same time, contrast-enhanced T1 mapping can be used to better quantify myocardial fibrosis and delayed enhanced inversion recovery imaging to identify different types of infiltrative cardiomyopathies [81]. Sometimes, as in AL amyloidosis, delayed gadolinium enhancement may have prognostic value along with serum biomarkers, while T1 has shown greater sensitivity than gadolinium in the early detection of amyloid deposits [82].

### 5.1. Cardiac Magnetic Resonance Imaging in Amyloidosis

As for the rest of the ICMs, for amyloidosis, CMR has a diagnostic and prognostic value. Compared to echocardiography, CMR has a better spatial resolution, allowing better measurement of the longitudinal and radial systolic function. These are strong arguments for considering CMR the gold standard in amyloidosis evaluation. Cine imaging offers basic diagnostic criteria, such as wall thickening, parameters of ejection fraction, and diastolic function, which allows differentiating between the types of amyloidosis [83]. In terms of prognostic potential, CMR can offer information about strain patterns, tricuspid and mitral annular plane systolic excursion, and pericardial or pleural effusion [84].

**Figure 5:**
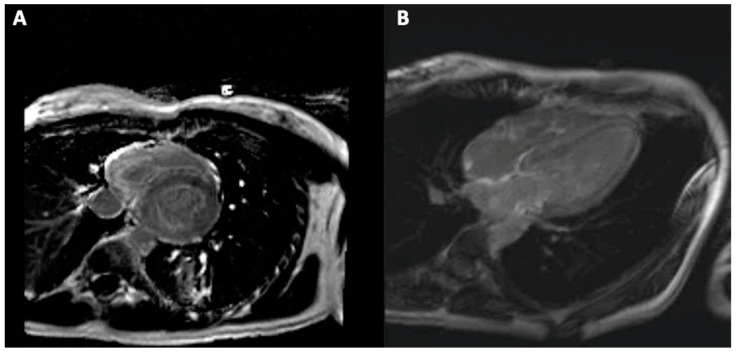
Biventricular subendocardial late gadolinium enhancement (**A**) and atrial late gadolinium enhancement (**B**) in a young patient with cardiac amyloidosis.

LGE is a valuable diagnostic tool in amyloidosis. However, the data regarding its prognostic value are controversial due to heterogeneous cardiac amyloidosis patterns, which generates differences in coding when using magnitude-only inversion recovery (MAG-IR). Moreover, in the early stages, the pattern is patchy, the presence of effusions determines artefacts, and arrhythmias’ occurrence makes the achievement of the images more difficult [85]. Even so, transmural LGE appears to determine higher mortality rates than the subendocardial one [86]. Its prognostic value has been certified for both AL and ATTR amyloidosis, while the differential diagnosis between these two is difficult to make based only on the LGE pattern [87]. Future research focuses on the right ventricle LGE as a prognostic factor in this population (Figure 5).

As amyloidosis consists of extensive interstitial infiltration, the T1 is usually raised. Additionally, the extracellular volume (ECV) is also increased, and it seems to correlate better with cardiac involvement and mortality than the native myocardial T1 [88]. The concomitant analysis of T1 and ECV can be a reliable tool in differentiating amyloidosis from other diseases that evolve with ventricular hypertrophy. Studies recommend the assessment of ECV as a means of identifying amyloidosis in the early stages, being particularly useful in follow-up and the evaluation of myocardial amyloid regression [89].

Recent studies illustrated the utility of ECV in distinguishing between AL and ATTR amyloidosis. By performing 1-(ECV × LV mass) and calculating the mean cell volume, an increase was illustrated only in ATTR amyloidosis. The histopathological explanation relies on cell hypertrophy added to the interstitial expansion which can be found in the ATTR form and is linked to better survival rates [90].

T2 mapping is correlated with myocardial edema and has limited benefit in amyloidosis. Nevertheless, recent studies have shown that T2 might increase in amyloidosis and is associated with a worse systolic function, which gives it a prognostic significance [91].

### 5.2. Cardiac Magnetic Resonance Imaging in Sarcoidosis

CMR has a 75–100% sensitivity and 76–78% specificity in diagnosing cardiac sarcoidosis, and it is thought to be more than twice as sensitive compared to the Japanese Ministry of Health and Welfare (JMHW) criteria for detecting cardiac involvement in general sarcoidosis [92]. This is due to its ability to detect myocardial edema, perfusion abnormalities, and scarring. In terms of inflammation evaluation, CMR, through its T2 weighted imaging and T2 mapping, has a significant contribution, even though the latter did not prove its superiority to PET [93]. T1 and T2 mapping were recently reported to be useful in the early detection of sarcoidosis as they identify the myocardial inflammation by directly relating to the altered magnetization properties [94].

For scarring evaluation, the method of choice is LGE. The common sarcoidosis lesions are localized in the septal, basal, and lateral segments of the left ventricle and papillary muscles with relative sparing of the subendocardium. The LGE pattern in sarcoidosis was correlated in different studies with patients’ prognosis in terms of the emergence of major cardiovascular events. For this reason, it has been suggested that CMR imaging should be used in the decision of cardioverter-defibrillator implantation [95]. Other studies identified LGE as an independent and strong predictor of all-cause mortality, sustained ventricular tachycardia episodes, or heart failure development [96]. LGE can also be used to monitor response to corticosteroids, the mainstay of treatment in cardiac sarcoidosis. One of the disadvantages of the LGE is that it cannot differentiate between active and chronic inflammation. In the early phase, wall edema and inflammation enhance wall thickness and motion abnormalities on cine images and increase T2 signal intensity of the involved area [97]. In the chronic phase, the walls are thin and dysfunctional. T1 and T2 could be successfully used for the early detection of cardiac involvement when LGE and left ventricle function were normal, with T2 weighted imaging being more specific for active inflammation and edema, and T1 being a better indicator of the onset of the disease.

Compared to FDG-PET, CMR has some advantages: lack of ionizing radiation exposure with low risk from gadolinium contrast in patients with GFR > 30 mL/min, higher spatial resolution, and better detection of small areas of fibrosis. It is important, however, not to forget that these two methods detect different pathologic features of cardiac sarcoidosis: inflammation with FDG PET and scarring with CMR, which is why some studies recommend the usage of combined techniques for a better evaluation of the patients in terms of myocardial function, the pattern of injury and sarcoidosis activity [98].

### 5.3. Cardiac Magnetic Resonance Imaging in Hemochromatosis

In hemochromatosis, CMR is advantageous in tissue characterization and myocardial iron overload (MIO) quantification. Because of myocardial iron accumulation, both T1 and T2 sequences are affected, but the validated method for MIO assessment is T2 mapping. Higher values of T2 are strongly correlated with hepatic and myocardial involvement, and lower ones may be related to higher risks of arrhythmias and heart failure. Because of its early detection capacity, CMR can guide therapy and follow-up, having an essential role in reducing mortality and morbidity [15].

## 6. Nuclear Imaging

Nuclear imaging modalities are useful in diagnosing patients with infiltrative cardiomyopathies, especially in those with amyloidosis and sarcoidosis, having the advantage of specific targeted molecular imaging. The most important techniques are positron emission tomography (PET) and single-photon emission computed tomography (SPECT), both of them having advantages and limitations [15]. The most important issue is that PET and SPECT are functional imaging techniques, as opposed to other imaging modalities, such as radiography or computed tomography, which are anatomical techniques. In addition, SPECT has the advantage of a robust, cheaper, and well-validated camera system, whereas PET has a high-spatial resolution, robust built-in attenuation correction, quantitative analysis, and low-patient radiation exposure [15,99]. Both of them form three-dimensional images, while scintigraphy, which also uses gamma cameras to detect internal radiation, forms two-dimensional images [99].

### 6.1. Nuclear Imaging in Amyloidosis

There are increasing data on the role of nuclear imaging techniques for the early identification and differential diagnosis of cardiac amyloidosis, especially transthyretin-related amyloidosis (ATTR). Radiolabelled SPECT phosphate derivatives, once used as bone-seeking tracers, were recently reported as a reliable method to localize amyloid deposits. There are several radiotracers evaluated for the diagnosis of cardiac amyloidosis, and they target different components altered in the heart: perfusion, metabolism, sympathetic innervation, or amyloid-deposits [100,101,102,103]. The most used SPECT tracer in Europe and Asia is 99mTc-diphosphonate (99mTc-DPD), while in USA it is technetium pyrophosphate (99mTc-PYP) [15,99]. It is essential to highlight that not all radiotracers are suitable for diagnosing cardiac amyloidosis, a possible explanation being the presence of microcalcifications. Thus, the microcalcifications that are more common in patients with ATTR amyloidosis than in those with AL amyloidosis cause a different myocardial retention of radiotracers [100]. One of the most important advantages of them is the avid uptake by ATTR and only minimal uptake with the light-chain (AL) amyloidosis subtype, providing one of the best non-invasive methods to differentiate these two subtypes [100]. By grading myocardial uptake in relation to rib uptake on SPECT and by quantifying radiotracer uptake using heart-to-contralateral lung ratio (H/CL), we may quantify the intensity of radiotracer uptake in the heart [104]. The current diagnostic criteria for patients with ATTR cardiac amyloidosis are a visual myocardial uptake ≥ than that in bone (specifically in the ribs) or a H/CL ratio of ≥1.5. Patients with a H/CL ratio of ≥1.6 have poor survival [105].

**Figure 6:**
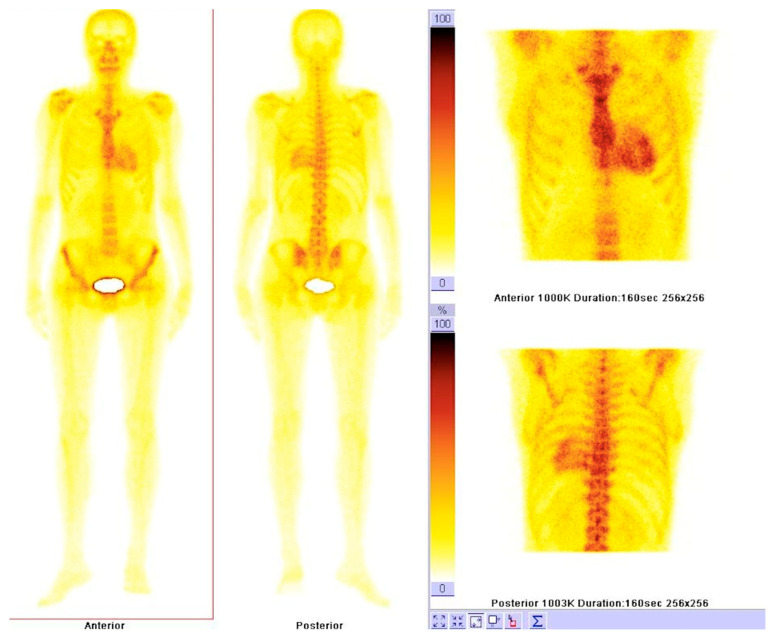
Bone scintigraphy with Technetium-99m hydroxymethylene diphosphonate illustrating abnormal myocardial uptake (Perugini grade 3) in a young woman with transthyretin amyloid cardiomyopathy.

Based on a simple visual scoring system of the delayed (3 h) planar image, Perugini and coworkers classified cardiac amyloid uptake in four grades: grade 0 (no cardiac uptake); grade 1 (mild cardiac uptake—less than in bone); grade 2 (cardiac uptake greater than that in bone, but uptake in bone remains clearly visible); and grade 3 (substantial cardiac uptake with a weak or no signal evident in bone) (Figure 6) [15,105].

A recent multicenter study demonstrated that bone-avid tracers have a 100% specificity and positive predictive value for the diagnosis of ATTR cardiac amyloidosis when used in combination without evidence of monoclonal protein [105]. Furthermore, using nuclear imaging, patients with ATTR cardiac amyloidosis can be diagnosed in an asymptomatic stage before increasing the wall thickness or reducing the electrocardiographic voltage [99,106]. In addition, patients with ATTR cardiac amyloidosis and a marked myocardial uptake of 99mTc-PYP have a poor prognosis due to the increased risk of major adverse cardiac events, acute heart failure and mortality [105,107].

Positron emission tomography is another nuclear imaging technique useful for the diagnosis of cardiac amyloidosis. There are several tracers that have been successfully used as PET tracers, facilitating the identification of amyloid deposits independently of the precursor protein. They are quantitative tools, and this allows the measurement of amyloid burden. Only small studies have demonstrated a higher myocardial retention index of 18F-florbetapir in patients with AL cardiac amyloidosis than in those with ATTR cardiac amyloidosis, and no significant uptake in healthy controls [108,109]. Similar findings were also observed in another small study that evaluated the myocardial retention of 18F-florbetaben in patients with cardiac amyloidosis compared with control patients with hypertension. They observed a higher myocardial retention in patients with AL or ATTR cardiac amyloidosis than in controls, this retention being inversely correlated with left ventricular global and right ventricular free-wall longitudinal strain [110].

Considering the results of these studies, the diagnosis of ATTR cardiac amyloidosis can be made with confidence when a patient presents with a clinical phenotype associated with echocardiographic or cardiac magnetic resonance findings consistent with amyloidosis, grade 2 or 3 tracer uptake in the heart on radionuclide bone scintigraphy, and the absence of detectable monoclonal immunoglobulin in the blood and urine [15]. Thus, only a minority of patients with ATTR cardiac amyloidosis will require endomyocardial biopsy, such as patients in whom a monoclonal immunoglobulin is detected, which raises the suspicion of AL amyloidosis [106].

Thus, nuclear imaging modalities are indispensable tools for early diagnosis, severity, and treatment planning in patients with cardiac amyloidosis. Compared with other imaging modalities, such as echocardiography or cardiac magnetic resonance, the most important advantages of nuclear imaging are the accessibility, simplicity of imaging, low cost, and also the specificity for cardiac ATTR amyloid deposits [99].

### 6.2. Nuclear Imaging in Sarcoidosis

Multimodality imaging has an important role in diagnosing sarcoidosis, in guiding the biopsy, and it can also evaluate the extent of the disease and help monitor patients under treatment. Cardiac involvement of sarcoidosis is an important prognostic factor and also increases the morbimortality of the patients. Nuclear imaging holds a pivotal role in the assessment of cardiac sarcoidosis [111]. Although [67Ga]-citrate scintigraphy is used as a major diagnostic criterion for cardiac sarcoidosis, it has a significantly lower sensitivity compared to [18 fluorine] fluoro-deoxy-glucose—positron emission tomography/computed tomography ([18F]FDG-PET/CT). This is the reason why [18F]FDG-PET/CT is nowadays the most common imaging tool for detecting myocardial inflammation.

Considering the inflammatory nature of cardiac sarcoidosis, PET is useful for diagnosis and treatment strategy plan, at both primary staging and patient follow-up. As inflammatory cells presented in active sarcoid lesions use glucose as an energy source, this will cause an increased [18F]FDG accumulation on PET/CT imaging [112]. Technical recommendations are very important, and before [18F]FDG-PET/CT, it is necessary that patients follow a high-fat–low-carbohydrate diet followed by prolonged fasting in order to suppress physiological myocardial glucose uptake [113].

Even though PET is more sensitive, it is also less specific than cardiac magnetic resonance (CMR), being more appropriate in patients with contraindications to CMR, in patients with inclusive findings on CMR, and when CMR is not available to monitor the response to therapy [15]. However, the two imaging modalities provide different significance of findings in patients with cardiac sarcoidosis. Thus, an increased [18F]FDG uptake represents active inflammation, whereas late gadolinium enhancement by CMR, indicates cardiac damage and scarring [112]. One of the most important advantages of FDG PET/CMR is the possibility to assess left ventricular wall function, the pattern of myocardial injury, and disease activity in a single scan [113]. In addition, serial [18F]FDG-PET/CT imaging can be used to evaluate the response to treatment, and to assess persisting inflammatory activity and metabolic response correlates with clinical outcomes. Studies reported a decreased [18F]FDG uptake in patients with cardiac sarcoidosis after corticosteroid or immunosuppressive therapies [114,115].

### 6.3. Nuclear Imaging in Hemochromatosis

In recent years, the role of [18F]FDG-PET imaging in diagnosis and treatment monitoring of inflammatory and infectious disease has been continuously growing. However, only a few clinical cases reported the role of [18F]FDG-PET imaging in the management of patients with hemochromatosis. To our knowledge, there are no studies about FDG PET/CT uptake of the heart in patients diagnosed with hemochromatosis, and only two studies reported about the liver uptake in patients with this pathology [116,117]. A recent study described a high liver uptake of [18F]FDG in a 64-year-old man with a history of non-Hodgkin diffuse B-cell lymphoma treated with chemotherapy, radiotherapy and stem cell transplant. After completion of treatment, [18F]FDG-PET/CT evaluation showed normal distribution. One month after, the patient came with a clinical and biological profile suggestive of hereditary hemochromatosis, and the PET/CT images performed as a follow-up for his malignancy, in the absence of tumor recurrence, revealed high glucose metabolism in the liver with a homogeneous pattern. After another 5 months, the patient was free of lymphoma, and the authors highlighted that the increased FDA uptake was secondary to liver damage caused by increased iron deposits secondary to intense hematological therapy [116].

In conclusion, to our knowledge, there is no evidence regarding the use of [18F]FDG-PET imaging in diagnosis and treatment monitoring of patients with cardiac hemochromatosis, and CMR remains the main imaging technique in this pathology. It allows quantification of myocardial iron overload and evaluation of RV and LV dimensions and functions.

In Table 1, we summarize the strength and the weakness of each imaging modality in the diagnosis and management of patients with infiltrative cardiomyopathies.

## 7. Implications of Multimodality Imaging in Monitoring Disease Progression and Treatment Response in Infiltrative Cardiomyopathies

### 7.1. Implications of Multimodality Imaging in Amyloidosis

The delay in recognizing cardiac amyloidosis is still a major impediment for the timely initiation of a targeted treatment. Observational studies have shown that if left untreated, subjects with systemic AL amyloidosis die within one year of diagnosis in the presence of cardiac involvement, while those with ATTR cardiomyopathy die within three years [118]. Considering the availability of new, effective disease-modifying agents for both types of amyloidosis, the usefulness of multimodality imaging in detecting and differentiating cardiac amyloidosis is greater than ever. Echocardiography only arouses suspicion of the presence of cardiac amyloidosis, but is neither sensitive nor specific. As highlighted in a systemic review and meta-analysis conducted by Brownrigg et al., CMR imaging is capable of diagnosing cardiac amyloidosis with high accuracy, but it requires nuclear imaging for further classifying the disease in ATTR or AL amyloidosis [119]. Therefore, implementing diphosphonate scintigraphy into the diagnostic algorithm of cardiac amyloidosis is of paramount importance as AL and ATTR amyloidosis claim for different therapeutic approaches. In AL amyloidosis, chemotherapy regimens consisting of melphalan, dexamethasone, and bortezomib, with or without autologous stem cell transplantation, are effective in decreasing free light chain production, the precursors of amyloid fibrils [119,120], while in patients with amyloidotic cardiomyopathy resulting from ATTR amyloidosis (either hereditary or wild-type), tafamidis is indicated in those with NYHA class I to III symptoms [121]. Patisirsan and inotersen should be considered only in patients with hereditary ATTR amyloidosis and polyneuropathy, with none of these two transthyretin gene silencers being recommended in hereditary ATTR cardiomyopathy without polyneuropathy or in wild-type ATTR cardiomyopathy [122].

For now, the ability of multimodality imaging in evaluating treatment response in cardiac amyloidosis has not been established, and no acknowledged definition of progression or response to therapy has been implemented in clinical practice. Survival, hospitalizations, functional capacity, quality of life, cardiac biomarkers, and different imaging techniques (including echocardiography, cardiac magnetic resonance imaging, and positron emission tomography) are currently used, but further studies addressing the utility of this approach are needed [121].

### 7.2. Implications of Multimodality Imaging in Sarcoidosis

A recent Danish nationwide cohort study of 11,834 patients with sarcoidosis and 47,336 control subjects underscored that sarcoidosis is associated with higher long-term risk of incident heart failure, higher mortality among the subjects who develop heart failure, and higher long-term risk of adverse cardiac outcomes, when compared to the background population, thus warranting for awareness and increased diagnostic performance [123].

Enhanced imaging technology has led to increasing recognition of cardiac involvement in systemic sarcoidosis, which typically manifests in atrioventricular conduction disturbances, ventricular arrhythmias, sudden cardiac death, and heart failure, and worsens the prognosis. There is a paucity of information regarding the best screening method for cardiac involvement in systemic sarcoidosis. Echocardiographic findings are non-specific and highly variable, but according to Mehta et al., when combining transthoracic echocardiography with Holter monitoring, electrocardiogram, and clinical history, the diagnosis of cardiac sarcoidosis can be attained in 100% of cases [124].

No pathognomonic findings for cardiac sarcoidosis can be revealed by cardiac magnetic resonance imaging, but the extent of late gadolinium enhancement, mainly located in the basal segments of the interventricular septum and lateral wall, typically in the epicardium and mid myocardium, emerged as an important prognostic factor [125,126,127].

Immunosuppressive agents are the mainstay of treatment in systemic sarcoidosis, but the effects of immunosuppression have not been systemically evaluated for cardiac sarcoidosis. According to an analysis performed by Cacoub et al., intravenous cyclophosphamide seems to correlate with a lower relapse rate in cardiac sarcoidosis [128]. Fluorodeoxyglucose positron emission tomography is a valuable imaging method of identifying active disease and therefore guiding immunosuppressive therapy [124].

Conclusively, subjects with cardiac sarcoidosis and LV dysfunction should be treated according to the current heart failure guidelines, with standard medical and device therapies or even heart transplantation [124].

### 7.3. Implications of Multimodality Imaging in Hemochromatosis

Long-term survival in hereditary hemochromatosis is mostly determined by the presence of liver involvement, but cardiovascular death can occur in about 20% of cases [129]. Cardiac magnetic resonance is the most powerful diagnostic and follow-up imaging tool in cardiac hemochromatosis, but transthoracic echocardiography has the advantage of being more approachable, providing instantaneous information regarding LV morphology and function [26].

Phlebotomy, chelation therapy with either parenteral deferoxamine or oral deferiprone and deferasirox, along with dietary interventions, epitomize the therapeutic armament against cardiac hemochromatosis [12].

## 8. Conclusions

Irrespective of the conflicting classification across the guidelines, ICMs embody the prototype of RCM. Cardiac amyloidosis, sarcoidosis, and hemochromatosis are common causes of ICM, demanding a high level of awareness and expertise to be diagnosed. By combining multimodality imaging techniques, a timely diagnosis can be attained, and valuable diagnostic and prognostic information can be acquired. Equally important, in ICM, the employment of multimodality imaging is of paramount significance in monitoring treatment response and improving outcomes via prompt disease-modifying interventions.

## Figures and Tables

**Table 1:** Strength and weakness of each imaging modality in the diagnosis and management of patients with infiltrative cardiomyopathies.

Multimo-dality Imaging	Cardiac Amyloidosis	Sarcoidosis	Hemochromatosis
**Echocardio-graphy**	**Strength:** widely available, LVH with bull’s eye pattern on strain imaging is a well-known red flag for CA. **Weakness:** lack of sensitivity and specificity.	**Strength:** first imaging screening method for CS. **Weakness:** lack of sensitivity in identifying early cardiac involvement and high variability of echocardiographic findings.	**Strength:** useful in screening and regular follow-up. **Weakness:** second-line imaging method, after CMR for the evaluation of cardiac hemochromatosis.
**Cardiac magnetic resonance**	**Strength:** able to establish the diagnosis of CA. **Weakness:** not capable of distinguishing between ATTR and AL amyloidosis.	**Strength:** allows an early identification of active inflammation and myocardial scarring. **Weakness:** there is no distinctive feature of CS on CMR.	**Strength:** CMR imaging (particularly T2 relaxation times) is the method of choice for assessing cardiac hemochromatosis, evaluating myocardial fibrosis and edema. **Weakness:** risk of gadolinium toxicity.
**Computed tomography**	**Strength:** enables myocardial characterization via LIE imaging as well as cardiac amyloid burden assessment via ECV quantification. **Weakness:** use of ionizing radiation and iodinated contrast.	**Strength:** useful in recognizing both cardiac and lung involvement, especially in subjects with metallic implants. **Weakness:** radiation-related risk and complications.	**Strength:** limited literature information available; might help evaluate cardiac function. **Weakness:** radiation exposure; provides static images, precluding dynamic analyses of left ventricular hemodynamics, filing or relaxation.
**Nuclear imaging**	**Strength:** useful in diagnosing ATTR cardiomyopathy beginning with early stages, eliminating the need of histological confirmation. **Weakness:** its diagnostic accuracy highly depends on the used radiotracers.	**Strength:** useful in monitoring disease activity and response to immunosuppressive therapy as well as in guiding biopsy. **Weakness:** less specific than CMR imaging, being recommended in subjects with contraindications to CMR.	No evidence available regarding the role of nuclear imaging in diagnosing, guiding therapy or monitoring disease evolution in cardiac hemochromatosis.

ATTR, transthyretin amyloid amyloidosis; CA, cardiac amyloidosis; CMR, cardiac magnetic resonance; CS, cardiac sarcoidosis; ECV, extracellular volume; LIE, late iodine enhancement; LVH, left ventricular hypertrophy.

## Data Availability

Not applicable.
